# Morphological differences in scoliosis curvatures as a cause of difficulties in its early detection based on angle of trunk inclination

**DOI:** 10.1186/s12891-022-05878-6

**Published:** 2022-11-02

**Authors:** Marek Kluszczyński, Dariusz Mosler, Jacek Wąsik

**Affiliations:** Faculty of Health Sciences, Jan Dlugosz University, Czestochowa, Poland

**Keywords:** Scoliosis, Early detection of scoliosis, Adams test, Vertebral rotation

## Abstract

**Introduction:**

The three dimensional deformation of the spine in scoliosis is specific for a given child with regard to the number and length of curvatures, their degree of rotation and the size of the curvature angle. Early diagnosis of scoliosis in a clinical examination according to the Adams test depends on the correlation between the angle of trunk inclination (ATI) and the Cobb angle and the adopted diagnosis criterion. The aim of the study was to demonstrate the need to adopt different diagnostic criteria for ATI depending on the age and location of scoliosis. Moreover, the observed differences in the ATI/Cobb correlation became the basis for the proposal to introduce the concept of low, medium and high-rotated of curvature to the clinical description of scoliosis.

**Materials and methods:**

The group consisted of 229 children who were first examined, aged 6 to 17 years, with an average age of -11.57 years (SD ± 3.26), with symptoms of idiopathic scoliosis. The correlation of the criteria for the diagnosis of scoliosis in the ATI 7° clinical trial with a Cobb angle of 10° three dimensional in the X-ray image was used to distinguish three types of curvature/scoliosis, i.e., low, medium and high rotation. The frequencies of each type were compiled for three age groups and three scoliosis locations. Moreover, the degree of vertebral rotation according to the Perdriolli (AVR) of curvature was correlated with the Cobb angle and ATI. A one-way logistic regression model was used to assess the effectiveness of scoliosis detection in children based on the measurement of the ATI angle alone and the measurement of both ATI and Cobb angles.

**Results:**

Low-rotated curves were most often found in the age groups of 6–9 and 10–12 years in 65.6% and 71.4% of patients, respectively (p < 0.05). Medium-rotated curvatures were most common in the age group of 13–17 years – 51.6%. With regard to the localization of scoliosis, the low-rotated curvatures were significantly more frequently (p < 0.05) found in the lumbar and thoracolumbar spine. Moreover, the univariate regression model for ATI showed that we could detect scoliosis best by taking the cut-off point of 5° and the mathematically determined Cobb angle was 9.5°. Patients with ATI ≥ 7° had significantly higher AVR values ​​than those with ATI < 7°, and the ATI/AVR correlation was of average strength.

**Conclusion:**

The specific morphology of the scoliotic curvature of the child’s spine may be manifested by the difference in the ATI/Cobb correlation depending on the location of the scoliosis and change with age. The curvatures of the scoliosis that form can be low, medium and high-rotated, and the low-rotated curvatures were most often found in the 6-9- and 10-12-year-old groups and in the lumbar and thoracolumbar section. To increase the rate of early diagnosis of scoliosis, the results suggest the need to adopt two ATI criteria for the diagnosis of scoliosis at screening, 5° for age of 6–12 years, and when asymmetry affects the lumbar and thoracolumbar section, and 7° for the remaining children.

**Supplementary Information:**

The online version contains supplementary material available at 10.1186/s12891-022-05878-6.

## Introduction

**Fig. 1:**
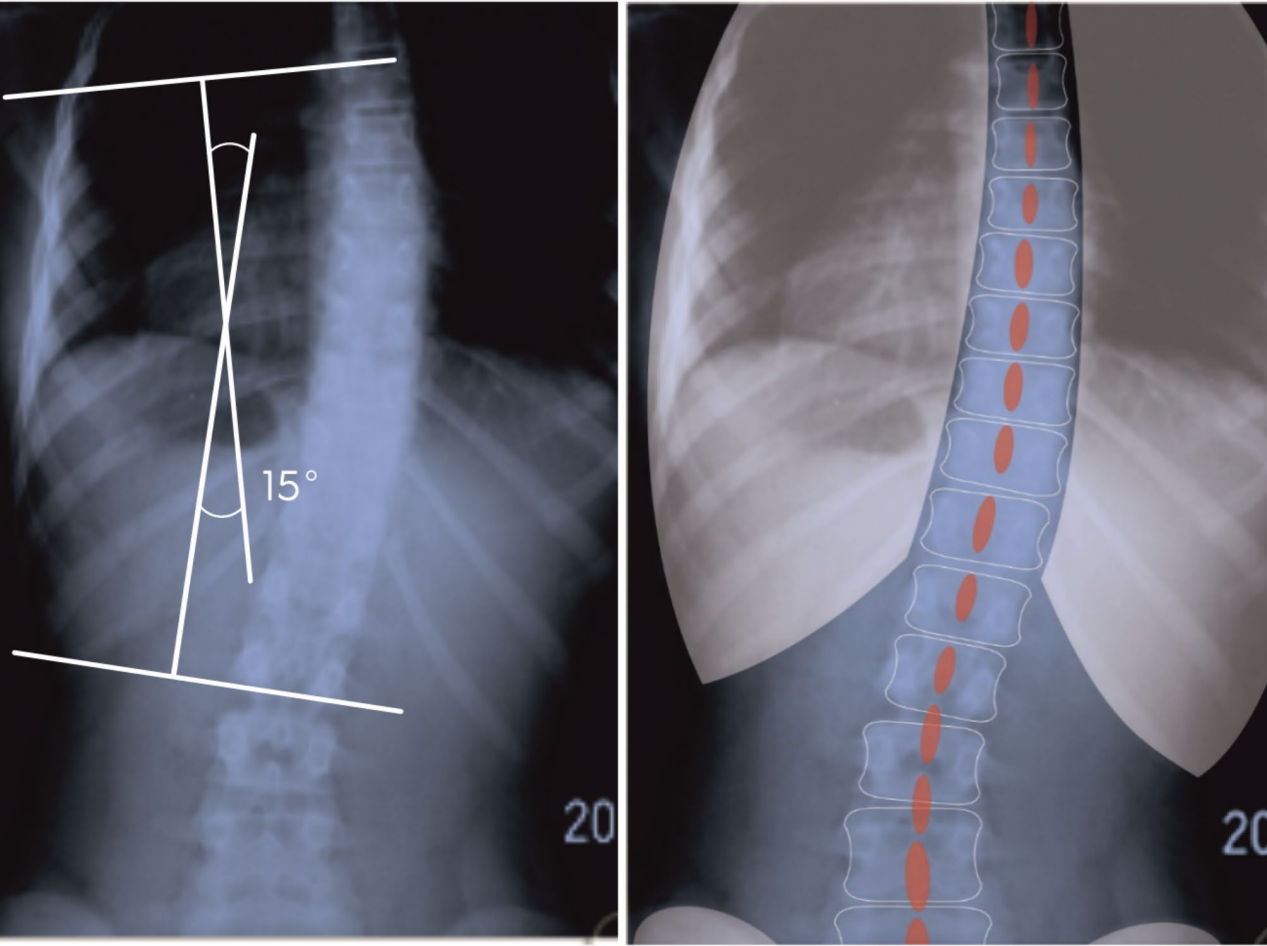
Low-rotated curvature/scoliosis. ATI is smaller than the correlation with Cobb angle would suggest. Scoliosis right thoracolumbar the Cobb angle – 15°, ATI – 4°

Early diagnosis of idiopathic scoliosis is a key factor determining the effectiveness of treatment [1, 2]. In a clinical trial, it was based on the ATI (Angle of Trunk Inclination) in the Adams test [1, 2]. Screening tests, when carried out diligently, play an indisputable role in the early diagnosis of scoliosis [3–6], but the proper diagnosis of scoliosis takes place in a doctor’s office [5]. The guidelines of the SOSORT and SRS scientific societies contain two criteria for the diagnosis of scoliosis, the ATI value ≥ 7° for screening tests and the ATI ≥ 5° or the so-called Hump ​​sum ATI ≥ 8° for specialized institutions [1, 5–7]. The decision to start X-ray diagnostics and treatment, in addition to the ATI criterion, should also take into account biological age, sex, family history, growth dynamics, body type and geographical location [1, 8]. The adopted ATI criterion is based on the alleged ATI/Cobb correlation, while the taken X-ray (gold standard) image reveals the truth about this correlation [9, 10]. It is then very common to find differences in the AVR/Cobb correlation between the spine sections, which also reflects the ATI/Cobb correlation. With the same ATI in the clinical examination, a different size of the Cobb angle is found on X-ray, and vice versa, with the same Cobb angle, a different ATI is found in individual sections of the spine [11–13]. Due to the changing morphology of the scoliotic curvature during the child’s growth, the interpretation of the ATI result, especially in screening tests, may be difficult, especially in less experienced diagnosticians [10]. When at the initial stage of scoliosis development there is a phenomenon of disproportionately low rotation of the vertebrae in relation to the size of the curvature angle, then, based on the ATI assessment, scoliosis may not be diagnosed. This scoliosis/curvature can be said to be low-rotated. Figure 1.

**Fig. 2:**
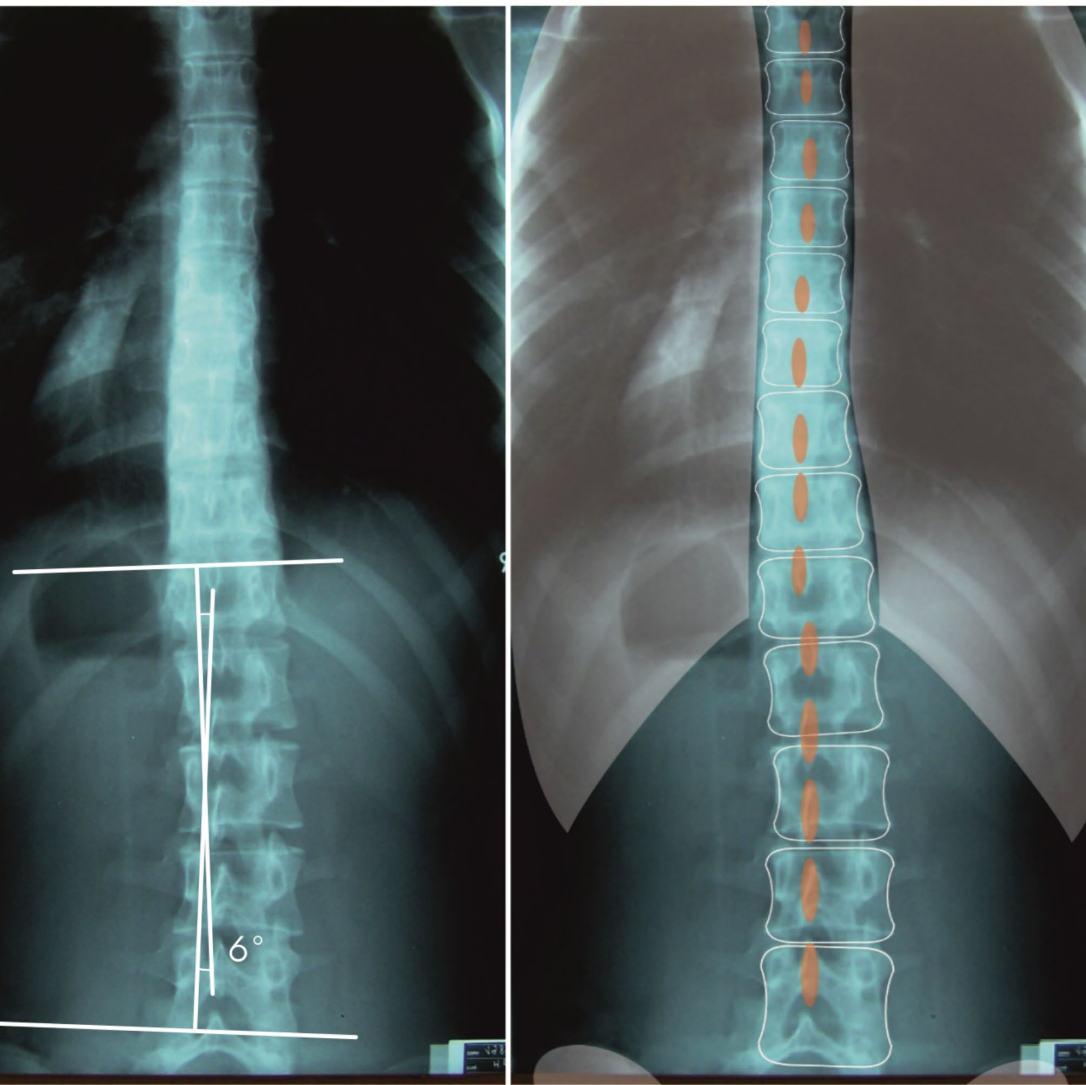
High-rotated scoliosis - ATI ≥ 7 ° and Cobb < 10 °. Scoliosis right lumbar Cobb 6°, ATI 8°

Conversely, when a significant rotation of the vertebrae ATI ≥ 7° is accompanied by a small Cobb angle < 10°, then the curvature/scoliosis can be defined as highly rotated. Figure 2.

**Fig. 3:**
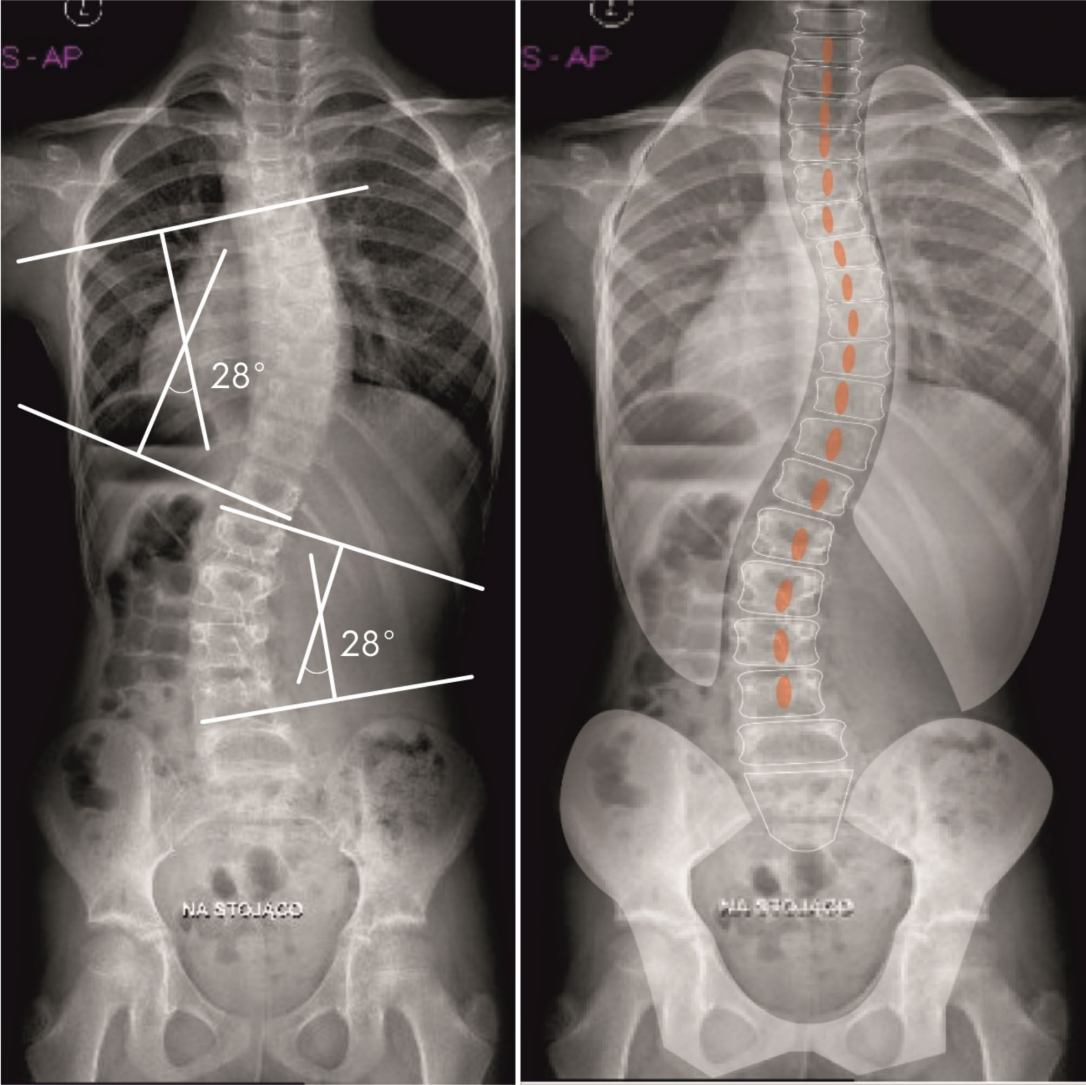
Medium-rotated scoliosis. ATI and the Cobb angle correlate with each other. Scoliosis right Th4-Th10 Cobb-28°, ATI 8°, left Th11-L4 Cobb 28°, ATI 9°

This type of curvature is much less common and is usually responsible for false positives. For the early diagnosis of scoliosis based on the ATI in the Adams test, it is ideal that the scoliosis develops with a high correlation that the size of the ATI angle correlates with the size of the Cobb angle. We are talking then about medium-rotated curvature/scoliosis, and then the diagnosis made on the basis of ATI is confirmed in the size of the Cobb angle on the X-ray. Figure 3.

The aim of the study was to demonstrate the need to adopt different diagnostic criteria for ATI depending on the age and location of scoliosis. Moreover, the observed differences in the ATI/Cobb correlation became the basis for the proposal to introduce the concept of low, medium and high-rotated curvature/scoliosis to the clinical description of scoliosis.

## Methods

The cohort study contains data from the medical records of children treated at the Troniny Rehabilitation Center in Klobuck, Poland. These patients were referred there by family doctors, paediatricians, orthopaedists or physiotherapists because of suspected scoliosis. The precondition for inclusion in the study was the availability of an up-to-date X-ray of the child’s spine during the first medical examination. The data of these patients were used to compare the values ​​of clinical parameters, in particular ATI, with the size of the Cobb angle and the vertebral rotation (AVR) on X-rays of untreated children. The tests were carried out by a rehabilitation specialist with 25 years of experience using the body posture assessment protocol used in the facility.

### Study participants

Out of 830 patients treated at the center, 229 children aged 6 to 17 were enrolled in the study; the mean age was 11.57 years (SD ± 3.26) and was comparable to the median value. The participants were assigned to three age groups, 6–9, 10–12 and 13–17 years old, according to their own assumptions dictated by clinical practice. The oldest group of children was the most numerous 41.7% (N = 95). In the group according to gender, girls accounted for the majority of 80.3% (N = 184). The selection of study participants was based on the following inclusion criteria: availability of a current (obtained in the last 3 months) radiograph of the child at the first clinical evaluation with symptoms of idiopathic scoliosis, Risser ≤ 3. Exclusion criteria were as follows: incomplete data in medical records, any previous treatment scoliosis as prior treatment, e.g., with orthopaedic braces, was considered a factor disturbing the assessment of variable, congenital vertebral deformities, neurological scoliosis, congenital defects, such as shortening of one limb more than 2 cm, genetic conditions, neurological diseases related to the locomotor organ, cardiovascular diseases, previous injuries or operations, neuromuscular disorders, and intellectual disability.

### Measurement

The ATI in the Adams test was assessed using a Bunnell’s scoliometer [5]. The severity of scoliosis was measured using the Cobb angle and the Perdriolli angle of rotation of the vertebrae (AVR) according to the SOSORT guidelines [2, 5, 11, 12].

To verify the accuracy of the ATI and Cobb angle measurements, an additional study was conducted by two physicians. One of them was the same person who conducted the main examination; another was a medical doctor with 15 years of experience in this field. Additional ATI and Cobb angle measurements were taken three times, day by day, in a group of 21 people who met the study criteria for age, gender distribution, and ATI and X-ray ranges, all of which were similar to the main study. These people were not treated with a brace or had any physical therapy between studies. The correlation coefficient between the class and the ICC within the observer was calculated; the latter for the ATI measurement ranged from 0.92 to 0.94, and for the Cobb angle from 0.96 to 0.98. The interobserver ICC for the ATI measurement was 0.92, and that for the Cobb angle was 0.96.

### Data analysis

For the analysis, three types of curvatures were distinguished based on possible clinical variants of the ATI/Cobb correlation (Figs. 1, 2 and 3):

- low-rotated curvature when ATI < 7° and Cobb ≥ 10°,
- medium-rotated curvature when ATI ≥ 7° and Cobb ≥ 10°,
- high-rotated curvature when ATI > 7° and Cobb < 10°.

The frequency of occurrence of particular types of curvatures was calculated for 3 age groups, 6–9, 10–12, 13–17 years, and three locations of curvature: lumbar, thoracolumbar and thoracic. Moreover, the size of the AVR was correlated with Cobb’s angle and ATI separately for age groups and location.

### Statistics

Data are presented as the arithmetic mean, standard deviation, median, minimum and maximum values, and percentages. Normality for individual variables was determined using the Shapiro-Wilk test. The nonparametric Mann-Whitney U test was used to compare the intergroup variables. The chi-square test or Fisher’s test was used to test the relationship between categorical variables. The Spearman correlation coefficient was used to study the relationship between ATI and X-ray variables. The adopted level of statistical significance was p = 0.05. One-way logistic regression models were used to assess the effectiveness of detecting scoliosis in children based on the measurement of the ATI angle alone and the measurement of both ATI and Cobb angles. R statistical package version 4.0.2 was used for all calculations and graphs.

### Trial registration

This study was approved by Jan Dlugosz University in Czestochowa Ethical Committee KE U/7/2021 and was approved by the Declaration of Helsinki. All the parents of the subjects were kept informed of the purpose and process of examination and subsequently gave their written consent before the study for participation and publication of results.

## Results

The characteristics of the 229 study participants are presented in Table 1. The mean ATI value was 6,76° (SD = 3.35°) and was slightly above the median value of 6°. The mean Cobb angle was 21.32° (SD = 11.45°) with a mean AVR of 18.34° (SD = 8.56°) and both values ​​were slightly above their medians. Table 1.

**Table 1:** Main characteristics of the patients

Variable	Parameter	Overall (N = 229)
Age division	6–9	27.9% (N = 64)
	10–12	30.4% (N = 70)
	13–17	41.7% (N = 95)
Gender	girls	80.3% (N = 184)
	boys	19.7% (N = 45)
ATI [°]- size of the angle of trunk inclination	N	229
	Mean (SD)	6.76 (3.35)
	Median (IQR)	6 (5–8)
	Range	2–25
X-ray [°]- size of the Cobb angle	N	229
	Mean (SD)	21.32 (11.45)
	Median (IQR)	18 (14–26)
	Range	3–75
Perdriolle angle [°]-angle of vertebrae rotation (AVR)	N	229
	Mean (SD)	18.34 (8.56)
	Median (IQR)	15 (10–25)
	Range	0–50

Patients with low rotated curvature significantly prevailed in the study group and accounted for 59.4% (N = 136), while patients with the medium-rotated curvature type accounted for 39.3% (N = 90). The type of highly rotated curvature was the least frequent, as it was found only in 1.3% (N = 3) of the respondents (Table 2).

**Table 2:** Frequencies of occurrence of the three scoliosis models in age groups

Age	Frequency in sample	Low-rotated 59.4% (N = 136)	Medium-rotated 39.3% (N = 90)	High-rotated 1.3% (N = 3)	^#^ p-value
**6–9**	27.9% (N = 64)	65.6% (N = 42)	32.8% (N = 21)	1.6% (N = 1)	**0.0112**
**10–12**	30.6% (N = 70)	71.4% (N = 50)	28.6% (N = 20)	0% (N = 0)	
**13–17**	41.5% (N = 95)	46.3% (N = 44)	51.6% (N = 49)	2.1% (N = 2)	

^#^ p value for Fisher’s test

When comparing the frequency of occurrence of particular types of curvatures in age groups, the occurrence of the type of low-rotated curvature/scoliosis was more frequent in the groups of children younger than 6–9 (65.6%) and 10–12 (71.4%) years old compared to the group of older children 13–17 years old (46.3%), where-medium rotated curvatures predominated (51.6%). High-rotated curvature/scoliosis occurred in a small percentage and constituted from 0 to 2.1% of children in the groups. Table 2.

With regard to the localization of curvature/scoliosis significant differences were also found in the frequency of the particular types of curvature/scoliosis. In the lumbar and thoracolumbar sections, low-rotated curves were significantly predominant, 70.9% (N = 39) and 64.3% (N = 54), respectively. On the other hand, in the thoracic segment, the most common curvatures were medium rotated i.e., 51.7% (N = 46). Table 3.

**Table 3:** Frequencies of occurrence of particular types of curvatures in relation to the location

Type curvature/scoliosis	Frequency in sample	Th (N = 89)	Th-L (N = 84)	L (N = 55)	^#^p-value
**Low-rotated**	59.4% (N = 136)	47.2% (N = 42)	64.3% (N = 54)	70,9% (N = 39)	**0.0202**
**Medium-rotated**	39.3% (N = 90)	51.7% (N = 46)	33.3% (N = 28)	29.1% (N = 16)	
**High-rotated**	1.3% (N = 3)	1.1% (N = 1)	2.4% (N = 2)	0% (N = 0)	

^#^ p value for Fisher’s test

**Fig. 4:**
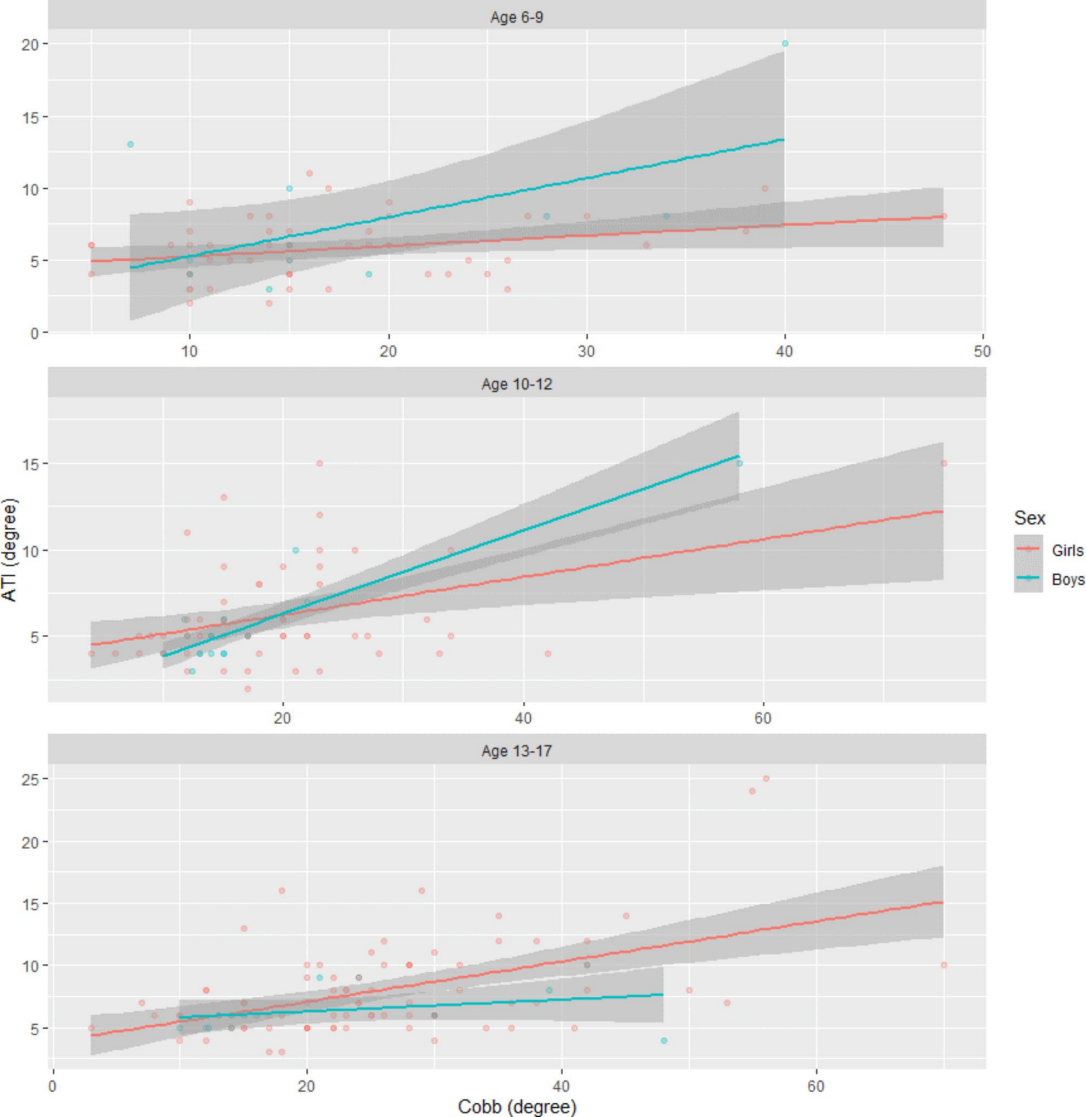
Spearman’s ATI/Cobb correlation coefficient by gender and age groups

The ATI/Cobb correlation for age and gender, calculated by Spearman’s coefficient in the study group, was low and ranged between 0.2507 and 0.5635, and the highest correlation was found in older age groups in girls (< 0.001) and in boys aged 10–12 years (p = 0.0185). Figure 4; Table 4.

**Table 4:** Spearman’s ATI/Cobb correlation coefficient by gender and age groups

Age	Girls		Boys	
	Spearman’s ATI/Cobb correlation coefficient	**p value** ^**#**^	Spearman’s ATI/Cobb correlation coefficient	**p value** ^**#**^
**6–9 age**	0.2507	0.0759	0.3862	0.1925
**10–12 age**	0.2714	**0.0493**	0.5635	**0.0185**
**13–17 age**	0.4379	**< 0.001**	0.4537	0.0894

^#^ p value for χ²test

The ATI/AVR correlation assessment with the nonparametric Mann-Whitney U test showed significantly (p < 0.001) higher AVR values ​​in patients with ATI ≥ 7° than in patients with ATI < 7°, which confirms the high diagnostic value of AVR. Table 5.

**Table 5:** AVR characteristics for ATI ≥ 7° and ATI < 7°.

Variable	Parameter	ATI ≥ 7° (N = 85)	ATI < 7° (N = 131)	p value^#^
Perdriolle angle [°]-angle of vertebrae rotation (AVR)	N	87	142	**< 0.001**
	Mean (SD)	22.47 (9.58)	15.81 (6.75)	
	Median (IQR)	20 (15–25)	15 (10–20)	
	Min-Max	0–50	5–35	

^#^ p value for the Mann–Whitney U test

The ATI/AVR correlation was significant both for the entire group and for each age group. It was positive and had mainly moderate strength (r = 0.29–0.35). However, no significant difference was found between the ATI/AVR correlation coefficients in the age groups. Table 6.

**Table 6:** Spearman’s ATI/AVR correlation coefficients for age groups

Age	Coefficient	LCI	UCI	p_value
Overall	0.379	0.256	0.485	**< 0.001**
6–9	0.290	0.048	0.495	**0.0201**
10–12	0.309	0.86	0.517	**0.0094**
13–17	0.348	0.162	0.519	**< 0.001**

The ATI/AVR correlation was similar for the different locations and was significant both for the entire group and for each localization. Correlations were positive and mainly of average strength (r = 0.38–0.40)(Table 7).

**Table 7:** Spearman’s ATI/AVR correlation coefficients for three locations of scoliosis

Section of the spine	Coefficient	LCI	UCI	p_value
Thoracic	0.396	0.176	0.573	**< 0.001**
Thoracolumbar	0.376	0.195	0.54	**< 0.001**
Lumbar	0.38	0.137	0.574	**0.0042**

Correlations between AVR/Cobb in the age groups were high, with the highest in the oldest group 0.91 (p < 0.001), and the correlation increased with the age of the child. Table 8.

**Table 8:** Spearman’s AVR/Cobb correlation coefficients for age groups

Age	Coefficient	LCI	UCI	p_value
Overall	0.886	0.838	0.918	**< 0.001**
6–9	0.834	0.733	0.902	**< 0.001**
10–12	0.85	0.757	0.912	**< 0.001**
13–17	0.913	0.858	0.948	**< 0.001**

When referring to the AVR/Cobb correlation dependent upon the location, statistically significant correlations were observed for each of the trunk rotation locations (p < 0.001). These correlations were positive and characterized by very high strength, especially in the thoracic segment (r = 0.905). Table 9.

**Table 9:** Spearman’s AVR/Cobb correlation coefficients for three locations of scoliosis

Section of the spine	Coefficient	LCI	UCI	p_value
Thoracic	0.905	0.823	0.951	**< 0.001**
Thoracolumbar	0.829	0.713	0.9	**< 0.001**
Lumbar	0.85	0.748	0.91	**< 0.001**

**Fig. 5:**
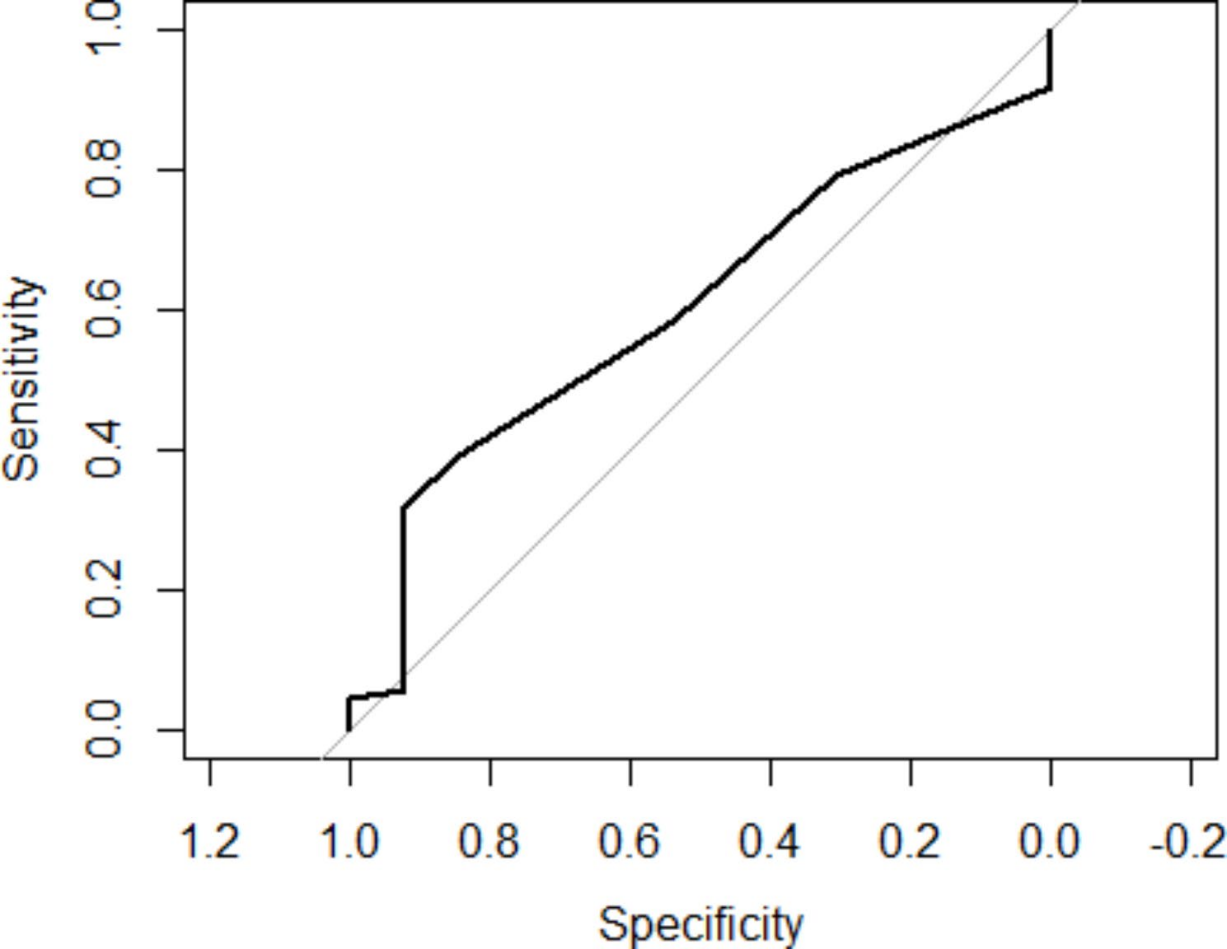
ROC curve for a diagnostic test based on the results of scoliosis for the entire group

The assessment of the effectiveness of scoliosis detection based on the ATI measurement in the univariate logistic regression model (ROC) showed the quality of AUC prediction at the level of 60%. Figure 5.

Using the ROC curve, the optimal cut-off point was determined for the occurrence of scoliosis, which was calculated for the entire group and separately for girls, which for both sets was 5.5°, which means that with this ATI criterion, the detection of scoliosis was the highest. Tables 10 and 11.

**Table 10:** Diagnostic test results based on the results of scoliosis for the entire study group

	Cut-off point	Sensitivity	Specifity	PPV	NPV	AUC
**Value**	5.5*°*	0.58	0.54	0.95	0.072	0.602
**95% Conf. Inter.**		0.33–0.81	0.46–1	0.95–1	0.062–0.14	0.46–0.74

**Table 11:** Results for the diagnostic test ego based on the outcomes of scoliosis for girls

	Cut-off point	Sensitivity	Specificity	PPV	NPV	AUC
**Value**	5.5°	0.61	0.58	0.95	0.095	0.666
**95% Conf. inter.**		0.37–0.82	0.5–1	0.95–1	0.083–0.19	0.56–0.78

## Discussion

Back asymmetry assessed in the Adams test is a recognized, pathognomonic symptom of scoliosis in the clinical assessment of a child’s posture [14]. The diagnosis of scoliosis is based on the putative ATI/Cobb correlation. The degree of vertebral rotation as an element of the three- dimensional deformation of the spine strictly determines the predictive value of ATI in the detection of scoliosis [6, 8–10].

Vertebral rotation and thus the aforementioned correlation may be influenced by many factors, among which the shape of the sagittal plane of the spine and the degree of ligament flexibility are of particular importance [15–18].

The structure and support-dynamic function of the pelvis and lower limbs also have an impact on the ATI value in the Adams test [19–23]. The three-dimensional spine in scoliosis is an extremely variable structure individually and difficult to assess through the ATI/Cobb correlation [24]. The subject of the study was the morphological differences in scoliosis curvature resulting from the correlation of the ATI in the clinical examination with the Cobb angle and AVR found on X-ray in relation to the child’s age and the location of the curvature [17, 24].

The high diagnostic value of the Adams test is confirmed by many publications [1, 2, 4–6]; however, researchers emphasize that the predictive value of the test is proportional to the degree of curvature [2, 9, 10]; moreover, it depends on the age of the child, [2, 6, 9, 24] of the spine in which the curvature begins, [24, 25] as well as the flexibility of the spine [15, 16] and from the investigator’s experience [25–29].

In the study, the state of undiagnosed scoliosis was defined by the low-rotating curvature, which can be defined by the formula ATI < 7°/Cobb ≥ 10°. The incidence of low-rotated curvature/scoliosis in the whole group was high (59.4%), especially in the younger groups, where it was 38% in the 6–12 year old age group. The predictive value of the ATI test is the lowest for the group of younger children and increases with age, which means that it is most difficult to recognize scoliosis in the Adams test at the beginning of its formation. Table 2.

Grivas et al. [10], on the basis of the ATI correlation analysis examined with the photogrammetric method with Cobb’s angle on X-ray, he also found weak correlation in the group of younger children. Similarly, other authors found that the ATI/Cobb correlation in 6–9 year old children with scoliosis in the lumbar region is not high [4, 8, 9]. It should be emphasized that the abovementioned morphological types of curvature may be characteristic of all curvatures of scoliosis [24, 29] or, what happens much more often, different types refer to its fragment, e.g., low rotation in the lumbar spine and medium or vice versa in the thoracic spine [5, 24]. Moreover, with growth and/or progression, the type of low-rotated curvature can change into medium or even high-rotated curvature [29–31].

When analysing the differences in the morphology of scoliosis curvatures in relation to the location in the study, it should be emphasized that low-rotated curvatures were significantly more frequent in the lumbar and thoracolumbar sections, which is consistent with the data in the literature [2, 10, 24]. Scoliosis of the first degree (10°-24° Cobb) located in the lumbar region, which develops in the type of low-rotated curvature, may be clinically manifested only by asymmetry of the waist angles, with slight ATI, while an X-ray often shows a significant Cobba ≥ 10 ° angle. Figure  1. Then, in a clinical trial with the Adams test, assuming the 7° criterion, scoliosis will not be diagnosed. When scoliosis is at an early stage of formation, the diagnosis in the Adams test will occur when the greatest curvature of the scoliosis (primary) is on medium-rotated, and Cobb values ​​≥10° will be accompanied by ATI ≥ 7. Figure  2.

In the study, the type of medium-rotated curvature was most often found in the group of the oldest children (13–17 years old) and in the thoracic location. Many studies confirm the observation that the highest ATI/Cobb correlation is most common in the thoracic spine and in older children [5, 6, 32].

The third type of curvature determining the ATI/Cobb correlation in which scoliosis can form is the highly rotated curvature, which is characterized by significant vertebral rotation (AVR) on X-ray and ATI ≥ 7° in the clinical examination and a small Cobb curve angle < 10°. Figure  3.

Scoliosis/curvature highly rotated was very rare in our study (1.3%). It is usually characterized by low progression dynamics [10, 24, 26], and in screening tests, it is responsible for some of the misdiagnosed scoliosis [10]. There was no significant correlation between the occurrence of this type of curvature in relation to age groups and the location of the curvature. Figure  3.

The study also looked at the AVR/ATI and AVR/Cobb correlations in the group to see if the ATI/Cobb correlation results presented correspond to the vertebral rotation found on X-ray. Figure  4; Table 4. The ATI/AVR correlation with respect to age groups and location was weak or moderate and increased significantly with increasing ATI angle and the age of the child. Similar results were presented by Amendt et al. [17], who, when assessing compliance with AVR/ATI found a weak correlation in the range of 0.32–0.46. These observations confirm the frequent discrepancy between the ATI results and the actual rotation of the vertebrae on the X-ray.

Krawczynski et al. [33], in a study of a similar design, found an ATI/Cobb correlation of 0.36 for the lumbar location and of 0.72 for the thoracic location; the corresponding ATI/AVR correlations were 0.60 and 0.71, respectively; and the corresponding AVR/Cobb correlations were 0.56 and 0.72, respectively [32]. These values ​​confirm the observations of our study.

In the study, referring to the comparison of the AVR/Cobb results, a high and very high correlation was found in all age groups, with an increasing tendency with age. In the aforementioned study, Amendt et al. [17] presented the AVR/Cobb correlation coefficient in the range from 0.48 to 0.70. Similarly, Korowesis et al. [25] found that this coefficient was in the range of 0.46–0.89. The results seem to be convergent, taking into account the possibility of measurement error [6, 28, 30, 34].

The evaluation of the effectiveness of scoliosis detection based on the ATI measurement presented in the ROC curve showed the quality of prediction measured by AUC at the level of 60%. The optimal ATI cut-off point for the occurrence of scoliosis was 5.5° both for the whole group and separately for girls, which means that with this ATI criterion, the detection of scoliosis was the highest. Adopting the ATI 5° criterion for younger children seems to significantly increase the detection of scoliosis in its early stage. Therefore, the results of the study led to the consideration of adopting two ATI criteria for the diagnosis of scoliosis in screening tests.

The proposal to distinguish three types of scoliosis curvatures describing the relationship between the rotation of the vertebrae and the lateral deflection of the spine is not intended to deny it but is intended to clarify the criteria in the context of early diagnosis of scoliosis, and the results of the study will be used for practical application in orthopaedic care for a child.

Weaknesses of the works is: The study was conducted in one institution, therefore to verify the results, multicentre studies should be carried out on a larger group of children.

## Conclusion

The specific morphology of the scoliotic curvature of the child’s spine may be manifested by the difference in the ATI/Cobb correlation depending on the location of the scoliosis and change with age. The curvatures of the scoliosis that form can be low, medium and high-rotated, and the low-rotated curvatures were most often found in the 6-9- and 10-12-year-old groups and in the lumbar and thoracolumbar locations. To increase the rate of early diagnosis of scoliosis, the results suggest the need to adopt two ATI criteria in screening: the ATI 5° criterion for children 6–12 years of age, particularly when asymmetry affects the lumbar and thoracolumbar section of the spine, and the remaining ATI 7° criterion.

## Electronic supplementary material

Below is the link to the electronic supplementary material.

Supplementary Material 1

Supplementary Material 2

## Data Availability

All data generated or analysed during this study are included in this published article and its supplementary information files.

## Declarations

### Statement

The authors of this study confirm that all methods were carried out in accordance with relevant guidelines and regulations.

## List of abbreviations

ATI: Angle of trunk inclination
AVR: Angle of vertebral rotation
IS: Idiopathic scoliosis
SRS: Scoliosis Research Society
SOSORT: Scoliosis Orthopedic and Rehabilitation Treatment
